# Complexity and Evolution

**DOI:** 10.3390/e25020286

**Published:** 2023-02-03

**Authors:** Tomas Veloz, Francis Heylighen, Olaf Witkowski

**Affiliations:** 1 Departamento de Matemática, Universidad Tecnológica Metropolitana, Las Palmeras 3360, Ñuñoa, Santiago 7800002, Chile; 2 Fundación para el Desarrollo Interdisciplinario de la Ciencia, la Tecnologia y las Artes, Santiago, Chile; 3 Centre Leo Apostel, Vrije Universiteit Brussel, Rue de la Strategie 33, 1060 Brussel, Belgium; 4 Facultad Ciencias de la Vida, Departamento Ciencias Biologicas, Universidad Andres Bello, Santiago 8370146, Chile; 5 Cross Labs, Cross Compass Ltd., Shinkawa 2-9-11-9F, Chuo-ku, Tokyo 104-0033, Japan; 6 Earth-Life Science Institute, Tokyo Institute of Technology, 2-12-1-IE-1 Ookayama, Meguro-ku, Tokyo 152-8550, Japan; 7 College of Arts and Sciences, University of Tokyo, 3-8-1 Komaba, Meguro-ku, Tokyo 153-8902, Japan

Understanding the underlying structure of evolutionary processes is one the most important issues of scientific enquiry of this century. In the twentieth century, scientific thinking witnessed the overwhelming power of the evolutionary paradigm. It not only solidified the foundations of diverse areas, such as cell-biology, ecology, and economics, but also fostered the development of novel mathematical and computational tools to model and simulate how evolutionary processes take place.

In addition to the application of the evolutionary paradigm and the discovery of the evolutionary features for processes of diverse nature, there is another interesting aspect which touches upon the emergence of novel evolutionary processes. Namely, the emergence of an evolutionary process requires a complex transition between a prior form where no evolutionary process is undergoing and a posterior form where the evolutionary process has been triggered.

Theoretical methods to describe the emergence of evolutionary processes require the consideration of complex systemic notions, such as self-organization, resilience, contextuality, among others. Therefore, complexity and evolution became intertwined notions: evolution not only leads to but also depends on the development of increasingly complex forms and functions.

In this Special Issue, we put together eight articles, mostly of interdisciplinary nature, that explore from recent advances in the modeling of complex systems, as well as of the increasing modeling power and growth of databases associated to evolutionary processes.

Our first article [1], by Peter Gardenfors, proposes that human causal reasoning, which is based on understanding the underlying forces that drive events, consists of an evolutionary process of mental representations about events that form the basis of language. The latter explains how language develops and complexifies over time.

Our second article [2], by Sergio Rubin and Michel Crucifix, argues that understanding the Earth as a complex system requires considering the Gaia hypothesis, which posits that the Earth is a complex system because it instantiates life at a planetary scale. The latter implies that the Earth’s complexity has formal equivalence to a self-referential system that inherently is non-algorithmic, meaning it cannot be surrogated and simulated in a Turing machine, and explores multiple consequences of this fact in relation to current planetary modeling.

Our third article [3], by Evo Busenniers, Francis Heylighen, and Tomas Veloz, a scenario for the self-organization of goal-directed systems explaining the origin of life is proposed. They utilize a framework called Chemical Organization Theory (COT) to demonstrate conditions under which reaction networks are able to form self-maintaining, auto-poietic organizations. The authors introduce new concepts, such as perturbation, action, and goal, and integrate them with existing concepts from cybernetics to explain goal-directedness. By following examples to test and refine these theoretical results, they suggest this could result in a realistic, step-by-step scenario for the evolution of goal-directedness, providing a theoretical solution to the question of the origins of purpose.

Our fourth article [4], by Yukio Pegio-Gunji and Daisuke Uragami, describes the concept of asynchronously-tuned elementary cellular automata (AT-ECA) and how they relate to active and passive updating and synchronous and asynchronous updating. It argues that the mutual tuning between these updating modes can be interpreted as a model for dissipative structure and can reveal complex properties in phase transitions from order to chaos. It claims that due to the asynchronous tuning, AT-ECA easily exhibits behavior at the edge of chaos, and that this property is referred to as the unfolded edge of chaos. The article also compares the computational power of AT-ECA with that of synchronous and asynchronous ECA, and finds that AT-ECA is more efficient.

Our fifth article [5], by Diederik Aerts and Lester Beltran, advances on previous work where they found that story texts exhibit a statistical structure that is Bose–Einstein rather than Maxwell–Boltzmann, which can be attributed to the indistinguishability of the meaning of words in different parts of the story, similar to indistinguishability in quantum mechanics. Here, they provide an explanation for this Bose–Einstein statistics in human language by showing that it is the presence of meaning in story texts that leads to the lack of independence characteristic of Bose–Einstein and providing evidence that words can be considered the quanta of human language. The researchers also show that meaning in text leads to decrease in entropy of the total text.

Our sixth article [6], by Tomasz Weron and Janusz Szwabiński, describes an agent-based model of opinion dynamics that examines the process of social polarization, where a community divides into opposing groups with contradictory opinions. It uses a double-clique topology with both positive and negative ties to the model of binary opinions. Individuals are subject to social pressure and conform to the opinions of their own clique and oppose those from the other one. The model also considers the chance of individuals acting independently. The results from Monte Carlo simulations and the mean-field approach lead to two main conclusions: a critical amount of negative relations is needed for polarization to occur, and independent actions actually support the process, unless their frequency is too high, in which case the system falls into total disorder.

Our seventh article [7], by Tomas Veloz and Pedro Maldonado, proposes a formal approach to investigate the emergence and evolution of worldviews using a metabolic system model. The article argues that none of existing cognitive models simultaneously provide high predictive capacity and a well-founded cognitive framework. The article proposes a general modelization of worldviews based on reaction networks, and a specific starting model based on species representing belief attitudes and species representing belief change triggers. The article claims that chemical organization theory combined with dynamical simulations can illustrate various features of how worldviews emerge, are maintained, and change in irreversible ways.

Our last article [8], by Lakshwin Shreesha and Michael Levin, describes an evolutionary simulation that studies the effects of different degrees of cellular competency on evolutionary dynamics. The simulation is based on minimal artificial embryogeny, in which virtual embryos are made of a single axis of positional information provided by cells’ structural genes, and the fitness of the embryos is determined by the monotonicity of the axial gradient. The simulation evaluates the evolutionary dynamics in two modes: hardwired development, where the genotype directly encodes phenotype and realistic mode, where cells interact before being evaluated by the fitness function. The results show that even minimal ability of cells to improve their position in the embryo results in better performance of the evolutionary search. It also suggests that increasing the behavioral competency masks the raw fitness encoded by structural genes, with selection favoring improvements to its developmental problem-solving capacities over improvements to its structural genome, suggesting strategies for bioengineering intelligent systems.

## References

[1] Gärdenfors P. (2021). Causal Reasoning and Event Cognition as Evolutionary Determinants of Language Structure. Entropy.

[2] Rubin S., Crucifix M. (2021). Earth’s Complexity Is Non-Computable: The Limits of Scaling Laws, Nonlinearity and Chaos. Entropy.

[3] Busseniers E., Veloz T., Heylighen F. (2021). Goal Directedness, Chemical Organizations, and Cybernetic Mechanisms. Entropy.

[4] Gunji Y.-P., Uragami D. (2021). Computational Power of Asynchronously Tuned Automata Enhancing the Unfolded Edge of Chaos. Entropy.

[5] Aerts D., Beltran L. (2022). Are Words the Quanta of Human Language? Extending the Domain of Quantum Cognition. Entropy.

[6] Weron T., Szwabiński J. (2022). Opinion Evolution in Divided Community. Entropy.

[7] Veloz T., Maldonado P. (2022). Modelling Worldviews as Stable Metabolisms. Entropy.

[8] Shreesha L., Levin M. (2023). Cellular Competency during Development Alters Evolutionary Dynamics in an Artificial Embryogeny Model. Entropy.

